# *E. coli* promotes human Vγ9Vδ2 T cell transition from cytokine-producing bactericidal effectors to professional phagocytic killers in a TCR-dependent manner

**DOI:** 10.1038/s41598-017-02886-8

**Published:** 2017-06-05

**Authors:** M. Barisa, A. M. Kramer, Y. Majani, D. Moulding, L. Saraiva, M. Bajaj-Elliott, J. Anderson, K. Gustafsson

**Affiliations:** 1 Infection, Immunity and Inflammation Program; UCL Great Ormond Street Institute of Child Health, 30 Guilford Street, London, WC1A 1EH United Kingdom; 2 Developmental Biology and Cancer Program, UCL Great Ormond Street Institute of Child Health, 30 Guilford Street, London, WC1A 1EH United Kingdom

## Abstract

γδT cells provide immune-surveillance and host defense against infection and cancer. Surprisingly, functional details of γδT cell antimicrobial immunity to infection remain largely unexplored. Limited data suggests that γδT cells can phagocytose particles and act as professional antigen-presenting cells (pAPC). These potential functions, however, remain controversial. To better understand γδT cell-bacterial interactions, an *ex vivo* co-culture model of human peripheral blood mononuclear cell (PBMC) responses to *Escherichia coli* was employed. Vγ9Vδ2 cells underwent rapid T cell receptor (TCR)-dependent proliferation and functional transition from cytotoxic, inflammatory cytokine immunity, to cell expansion with diminished cytokine but increased costimulatory molecule expression, and capacity for professional phagocytosis. Phagocytosis was augmented by IgG opsonization, and inhibited by TCR-blockade, suggesting a licensing interaction involving the TCR and FcγR. Vγ9Vδ2 cells displayed potent cytotoxicity through TCR-dependent and independent mechanisms. We conclude that γδT cells transition from early inflammatory cytotoxic killers to myeloid-like APC in response to infectious stimuli.

## Introduction

γδT cells express a T cell receptor (TCR) composed of γ and δ chains, and constitute 1–15% of human peripheral blood mononuclear cells (PBMC); and up to 40% of intraepithelial lymphocytes in epithelial linings^1^. A broad categorization in humans is defined by Vδ chain expression, constituting Vδ1^+^, Vδ2^+^ and Vδ1^−^Vδ2^−^ subsets. Human γδT cells possess high functional plasticity encompassing cytokine production, innate-like cytotoxicity, wound-healing, immunoregulation and professional antigen presenting cell (pAPC) properties^2^. Evidence suggests that the predominant human peripheral γδT cell subset, with a Vγ9Vδ2 TCR, is involved in immuno-surveillance of stress signals emanating from endogenous (e.g. tumor cells) and microbial pyrophosphates (e.g. infected cells)^3^.

Significant increase in systemic and mucosal γδT cells is seen in several acute infectious diseases. This effect is particularly pronounced in systemic bacterial and parasitic infections, which include *Brucella*, *Streptococci*, *Coxiella*, *Listeria*, *Francisella*, *Shigella*, *Leptospira*, *Plasmodium* and *Mycobacterium* infections amongst others^4–13^. While the functional phenotype of *in vivo* expanded γδT cells remains poorly examined, recorded observations indicate an activated phenotype, as evidenced by high cell surface levels of CD69, and significantly elevated expression of MHC class II (e.g. HLA-DR) and CD86^11, 12, 14–16^. The presence of CD69^pos^HLA-DR^pos^ γδT cells in sepsis and systemic inflammatory response syndrome correlates negatively with mortality^15, 17^. Although studies have documented *ex vivo* expansion of primary γδT cells upon PBMC exposure to infectious agents, detailed information on phenotypic cell changes is lacking^4, 18–21^.

The *in vivo* observations of γδT cell expansion in clinical infectious disease, and the *ex vivo* exploration of human γδT cell pAPC function and phagocytosis by Brandes *et al*. and our own laboratory^22–24^, prompted us to investigate how Gram-negative bacteria may modulate the plasticity of this unique T cell population. We hypothesized that the γδT cell response within PBMC to whole, freshly UV-irradiated *E. coli* reflects events that occur during a systemic infection. *E. coli*, a causative agent of human sepsis and bacteremia, expresses phosphoantigens that are documented potent activators of peripheral Vγ9Vδ2 γδT cells^19, 25^. *E. coli* is, moreover, a human intestinal commensal and frequent cause of infections at a site highly populated by γδT cells. We therefore examined γδT phenotype and function in response to acute *E. coli* exposure and in response to re-exposure of expanded cells. Responses were compared to zoledronic acid, a drug, which is a known stimulator of Vγ9Vδ2 γδT cell expansion via accumulation of endogenous pyrophosphates^26^. In response to *E. coli*, peripheral human Vγ9Vδ2 γδT cells transitioned from early Th1-like, cytotoxic responders to cytotoxic, phagocytic pAPCs. This model allowed us to address which of these effector functions are dependent on the TCR.

## Results

### Zoledronate-expanded γδT cells take up IgG-opsonized 1.0 μm beads and *E. coli*

**Figure 1:**
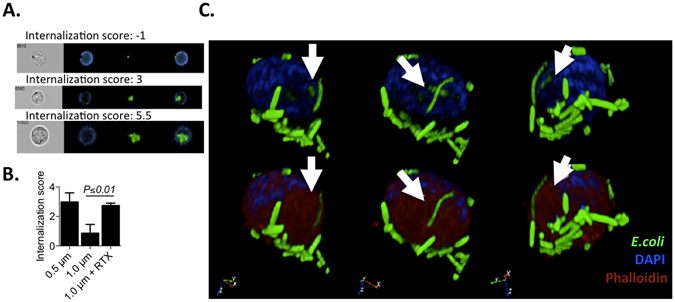
Zoledronate-expanded γδT cells take up IgG-opsonized 1.0 μm beads and *E. coli*. (**A**) 14 day zoledronate-expanded γδT cells (n = 3) were incubated with IgG-opsonized or non-opsonized polystyrene beads or IgG-opsonized *E. coli* for 60 min, and analyzed for internalized material. γδT cell uptake of beads was assessed with an internalization score generated via ImageStream analysis. Representative donor data is shown, with γδTCR in blue and beads in green. (**B**) PBMC were cultured for 60 min with non-opsonized 0.5 μm and 1.0 μm beads, as well as IgG (Rituximab; RTX)-opsonized 1.0 μm beads. PBMC were then stained for ImageStream analysis; internalisation scores are shown for γδT cells. (**C**) FACS-purified γδT cells were stained with phalloidin (red), DAPI (blue), incubated with opsonized, GFP-expressing *E. coli*, and analyzed via confocal microscopy. Representative data is shown of a single cell in 3D-rotation with or without phalloidin. Internalised *E. coli* is indicated with white arrows.

Phagocytosis, a crucial component of APC function is defined as receptor and actin-polymerization-dependent uptake of material >0.5 μm in size^27^. We have previously reported limited phagocytosis by freshly-isolated peripheral γδT cells, resulting in processing and presentation on MHC class I and II of associated peptides^23, 24^. Herein, we explored the impact of cell expansion on γδT cell phagocytic capacity in detail. 14 day zoledronic acid (zoledronate)-expanded γδT cells were co-cultured with protease-activated DQ-Green fluorescent, bovine serum albumin (BSA)-labeled polystyrene beads (0.5 μm or 1.0 μm in size), with or without IgG opsonization. DQ-Green, BSA-labeled beads have been employed previously as an indicator of phagosome maturation and antigen processing in macrophages^28^. Internalization of fluorescing, i.e. protease-exposed, beads was quantified using an ImageStream internalization score (Fig. 1A). Expanded γδT cell incubation with non-opsonized beads revealed significant uptake of 0.5 μm, but not 1.0 μm beads. Opsonization with Rituximab (monoclonal, chimeric human-mouse IgG against CD20) significantly enhanced 1.0 μm bead uptake - to a level statistically indistinguishable from the uptake of beads 0.5 μm in size (Fig. 1B). Internalization scores indicated that ~9% of γδT cells associated with opsonized beads, of which ~86% showed internalization. The observation that a significant portion of expanded γδT cells internalize opsonized beads into a protease-rich environment prompted us to investigate γδT cell uptake of bacteria, such as *E. coli*. Confocal microscopy allowed detection of whole and partially-degraded *E. coli* in the interior of zoledronate-expanded γδT cells incubated with IgG-opsonized, GFP-expressing *E. coli* (Fig. 1C). As exemplified in Fig. 1C, virtually all γδT cells within the field of vision were associated with multiple adherent *E. coli*, whereupon only a minor fraction of the bacteria were found to be intracellular (data not shown).

### *E. coli*-expanded, but not freshly-isolated γδT cells, phagocytose IgG-opsonized *E. coli*

**Figure 2:**
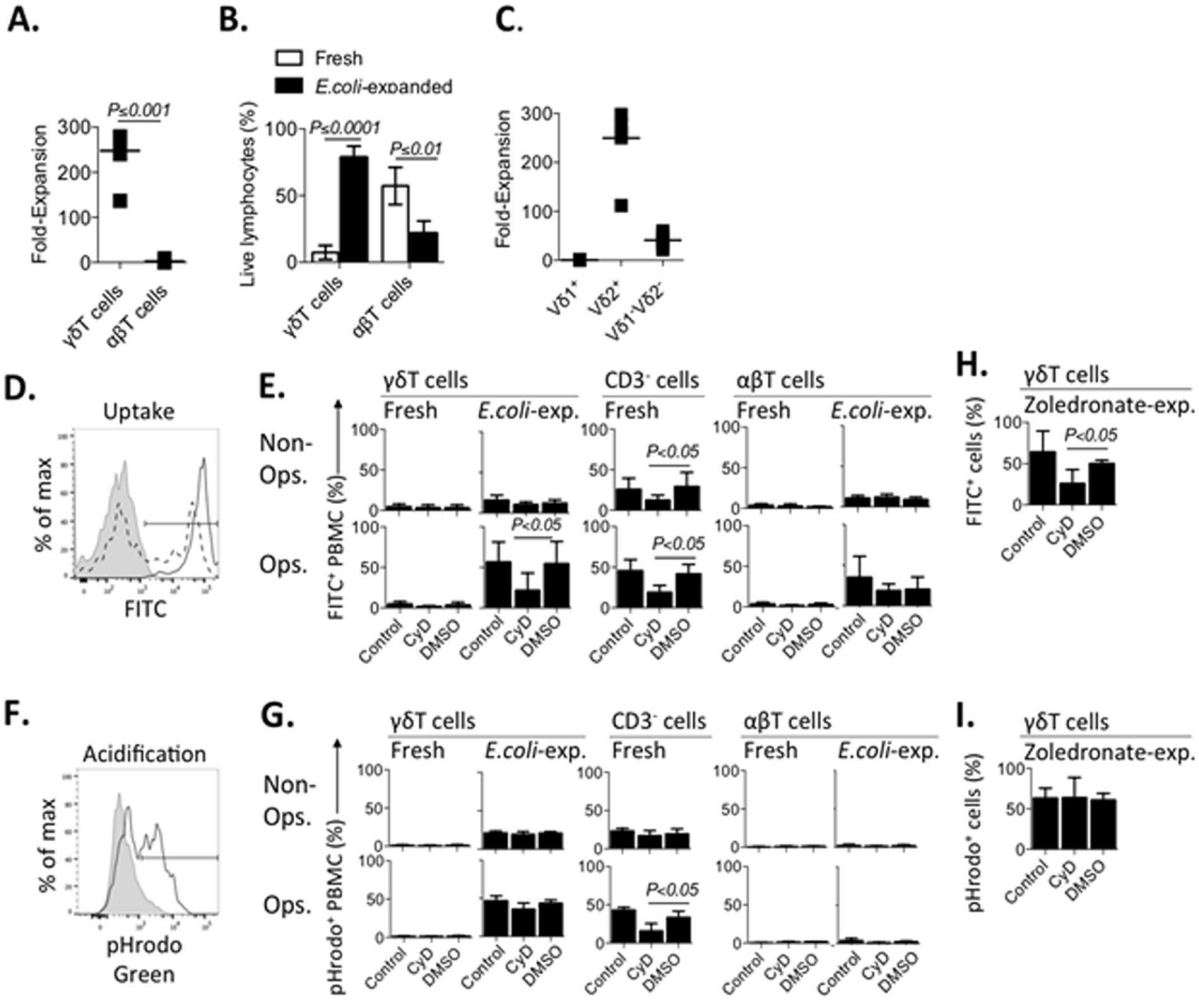
*E. coli*-expanded, but not fresh, γδT cells phagocytose IgG-opsonized *E. coli*. Freshly-isolated PBMC (n = 5) were analyzed for *E. coli* uptake immediately or stimulated with *E. coli*, left to expand for 14 days, and examined on day 14. To determine uptake, PBMC were incubated with IgG-opsonized or non-opsonized, fluorescently-labeled *E. coli* for 60 min. PBMC were pre-cultured with normal media (control), Cytochalasin D (CyD) or DMSO. (**A**) Fold-expansion of γδT and αβT cells, assessed by FACS and Trypan Blue exclusion, was compared in 14 day *E. coli-*stimulated PBMC. (**B**) PBMC were compared via FACS for γδT and αβT cell content of total live lymphocytes. (**C**) Fold-expansion in response to *E. coli* over 14 days was compared between γδT cell subsets. (**D**) PBMC were incubated with FITC-labeled *E. coli* and quenched post-culture with Trypan Blue. Shown are representative stains, gated on γδT cells: i) non-quenched co-culture indicating total FITC fluorescence (black, solid, unshaded), ii) quenched co-culture indicating intracellular FITC fluorescence (black, dotted, unshaded), iii) co-culture with non-FITCylated *E. coli* (gray, shaded). (**E**) The proportion of FITC^pos^ PBMC was examined. (**F**) PBMC were incubated with pHrodo-labeled *E. coli*. Shown are representative stains: i) PBMC, gated on γδT cells, co-cultured with pHrodo-*E. coli* (black, solid, unshaded), ii) pHrodo-*E. coli* only control (gray, shaded). (**G**) The proportion of pHrodo^pos^ PBMC was examined. (**H**) Uptake of IgG-opsonized FITC-*E. coli* and (**I**) acidification of IgG-opsonized pHrodo-*E. coli* was examined in 14 day zoledronate-expanded γδT cells.

We next quantified the uptake of IgG opsonized *versus* non-opsonized *E. coli* by freshly-isolated *versus E. coli-*expanded γδT cells. Freshly-isolated PBMC from healthy laboratory donors were co-cultured with UV-irradiated *E. coli* and left to expand for 14 days. Expansion resulted in a marked increase in CD3^pos^ cells (Fig. S2A), with a preferential (>200-fold) expansion of γδT cells (Fig. 2A,B). It was interesting to note that a population of αβT cells persisted with minimal expansion (Fig. 2B). Vδ2^+^ γδT cells displayed the highest rate of expansion (~250-fold), followed by Vδ1^−^Vδ2^−^ cells (~40-fold) and Vδ1^+^ γδT cells, which instead contracted (Fig. 2C). Donor-matched, parallel expansions of PBMC in IL-2 media with zoledronate or *E. coli* induced similar rates of expansion of subsets (Fig. S2B). Of note, IL-2 media alone failed to induce expansion of γδT or αβT cells (data not shown).

To measure phagocytosis, freshly-isolated or expanded PBMC were incubated with IgG-opsonized or non-opsonized FITCylated-*E. coli* for 60 minutes, and analyzed via Trypan Blue quenching and flow cytometry (Fig. 2D). Freshly-isolated γδT and αβT cells failed to show notable bacterial uptake; in contrast ~25% and ~45% of freshly-isolated CD3^neg^ PBMC (predominantly monocytes) internalized non-opsonized and opsonized *E. coli*, respectively; both processes were found to be sensitive to cytochalasin D (CyD), an inhibitor of actin polymerization (Fig. 2E). More than 50% of *E. coli-*expanded γδT cells took up opsonized *E. coli* in a CyD-sensitive manner. Interestingly, a subpopulation (mean 35%) of the residual αβT cells following *E. coli* expansion also took up opsonized *E. coli*, but this phenomenon was not significantly inhibited by CyD (Fig. 2E).

Phagocytosis promotes fusion of the phagosome with the lysosomal compartment. To determine whether *E. coli* uptake by *E. coli-*expanded γδT cells resulted in bacterial acidification (implying phagolysosome formation), freshly-isolated and 14 day expanded PBMC were co-cultured for 60 minutes with IgG-opsonized pH-sensitive pHrodo-*E. coli* (Fig. 2F)*. E. coli-*expanded γδT cells, but not αβT cells or freshly isolated γδT cells, showed notable acidification of *E. coli*, which increased further upon opsonization. As a positive control, freshly isolated CD3^neg^ PBMC (largely monocytes) also acidified non-opsonized and opsonized *E. coli*. Only acidification by CD3^neg^ PBMC was CyD-sensitive (Fig. 2G). A similar degree of bacterial uptake, acidification and CyD-sensitivity was observed between *E. coli* and zoledronate-expanded γδT cells (Fig. 2H,I). It remains unclear as to why γδT cell bacterial uptake but not acidification appeared CyD-sensitive. One possible explanation may be the difference in bacterial preparations employed, as fresh exponentially-grown *E. coli* were irradiated just prior to uptake studies whilst lyophilized, *E. coli-*pHrodo conjugates were utilized to examine acidification. Lyophilisation may lead to bacterial acquisition of a spherical rather than rod shape, with consequential changes to the involvement of the actin cytoskeleton in the uptake process^29, 30^. Nonetheless, this series of experiments provides evidence for the first time that, upon expansion, γδT cells can phagocytose and direct bacteria to an acid-rich environment. Interestingly, the magnitude of bacterial acidification varied between expanded γδT cells and freshly-isolated CD3^neg^ PBMC (Fig. S3), suggesting cell-specific pathways may be involved in bacterial uptake and processing.

### *E. coli* and zoledronate-expanded γδT cells phagocytose *E. coli* in a TCR-dependent manner

**Figure 3:**
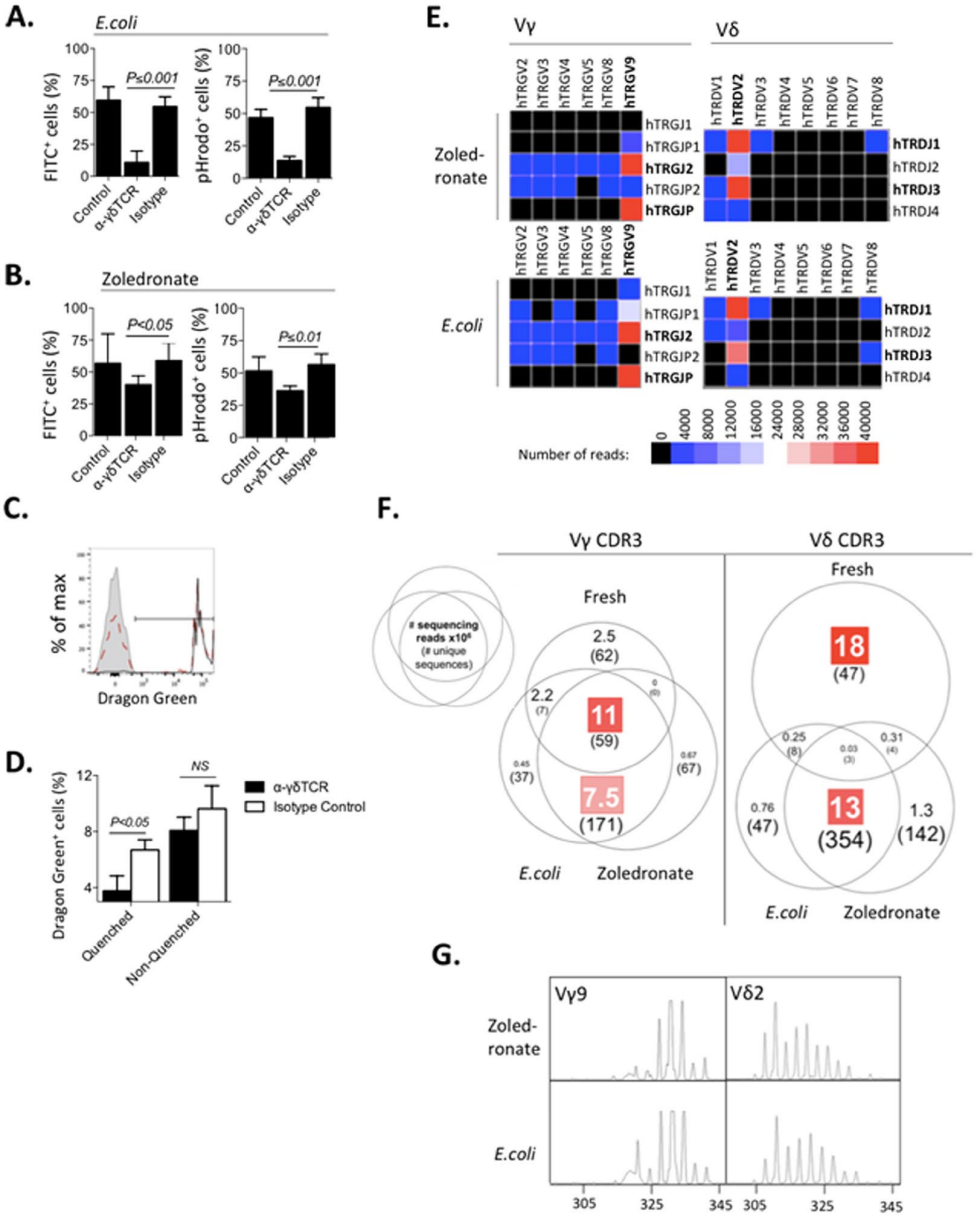
*E. coli* and Zoledronate-expanded γδT cells phagocytose *E. coli* with similar dynamics in a TCR-dependent manner. Freshly-isolated PBMC (n = 5) were expanded with UV-irradiated *E. coli* or zoledronate for 14 days, and examined for *E. coli* uptake on day 14. To determine uptake, PBMC were incubated with IgG-opsonized *E. coli* for 60 min. PBMC were pre-cultured with normal media (control), anti-γδTCR mAb (clone: B1) or isotype-matched control. The effect of pre-culture with anti-γδTCR mAb on phagocytosis was examined; data is shown for (**A**) *E. coli-*expanded or (**B**) zoledronate-expanded γδT cells, incubated with FITC-*E. coli* or pHrodo-*E. coli*. (**C**) PBMC were incubated with opsonized green fluorescent beads and quenched post-culture with Trypan Blue. Shown are representative stains, gated on γδT cells: i) non-quenched co-culture indicating total bead fluorescence (black, solid, unshaded), ii) quenched co-culture indicating intracellular bead fluorescence (red, dotted, unshaded), iii) cells alone (gray, shaded). (**D**) The effect of anti-γδTCR mAb was examined on γδT cell uptake of opsonized bead in quenched and non-quenched samples (n = 3). (**E**) TCR sequencing was carried out on *E. coli* or zoledronate-expanded and fresh non-expanded γδT cells. Representative heat maps of one donor *E. coli* and zoledronate-expanded γδT cell Vγ (VG), Vδ (VD) and Vj (VJ) chain reads are shown. (**F**) The number of shared TCR CDR3 sequences was tallied and compared in *E. coli* or zoledronate-expanded and fresh unexpanded γδT cells. A comparison of the number of sequencing reads shared as well as the number of unique sequences in each category is shown for both Vγ and Vδ TCR chains. For all samples, total depth of sequencing was equivalent. Only sequences with more than 5000 reads were tallied to select for the commonest clones. (**G**) TCR CDR3 spectratyping was carried out for *E. coli* or zoledronate-expanded Vγ9Vδ2 T cells. Representative spectratypes of one donor are shown.

As *E. coli* and zoledronate-expanded γδT cells phagocytosed opsonized bacteria with similar dynamics, we hypothesized that both agents may employ overlapping signaling pathways in these processes. To examine the importance of the TCR in γδT cell phagocytosis, *E. coli* or zoledronate-expanded γδT cells were cultured with anti-γδTCR mAb (clone: B1), isotype-matched control mAb of known non-*E. coli* specificity (clone: MG1-45) or media alone, prior to co-culture. Both uptake and acidification of opsonized bacteria by *E. coli*-expanded γδT cells were highly sensitive to TCR inhibition whilst zoledronate-expanded γδT cells were less sensitive (Fig. 3A,B).

To further investigate the role of the TCR in γδT cell phagocytosis, expanded PBMC were incubated with IgG-opsonized green fluorescent beads for 60 minutes, and analyzed via Trypan Blue quenching and flow cytometry, with or without γδTCR blocking (Fig. 3C). Uptake of opsonized beads by expanded γδT cells was significantly inhibited in the presence of anti-γδTCR mAb (Fig. 3D). Bead adherence, as measured in non-quenched PBMC-bead co-culture, was not affected by blocking of the TCR.

The TCR CDR3 regions of (a) freshly-isolated unexpanded, (b) *E. coli*-expanded and (c) zoledronate-expanded γδT cells from one representative donor were sequenced. *E. coli* and zoledronate-expanded γδT cells showed significant overlap of their Vγ and Vδ CDR3 sequences; both expansions resulted in a predominantly Vγ9Vδ2 γδT cell population (Fig. 3E). CDR3 homology and frequency in selected sequences that represented 5000 or more CDR3 reads were investigated (Fig. 3F). The most prominent group in Vγ chain CDR3 sequences was shared between all three subsets, suggesting a largely conserved expansion. The other prominent group was shared between *E. coli* and zoledronate-expanded γδT cells, with only low counts exclusive to either group. ~80–90% overlap in the Vγ and the Vδ chain CDR3 regions was observed between the two expansion protocols. Notably, the single exclusive group of Vδ chains was found in freshly isolated γδT cells, indicating a significant shift in Vδ chain CDR3 repertoire post expansion. Striking homology in CDR3 spectratypes was observed in the Vγ9 and Vδ2 chains of *E. coli* and zoledronate-expanded γδT cells, confirming CDR3 overlap (Fig. 3G).

### γδT cell acquisition of phagocytic capacity is concurrent with sustained upregulation of cell surface HLA-DR and CD86

**Figure 4:**
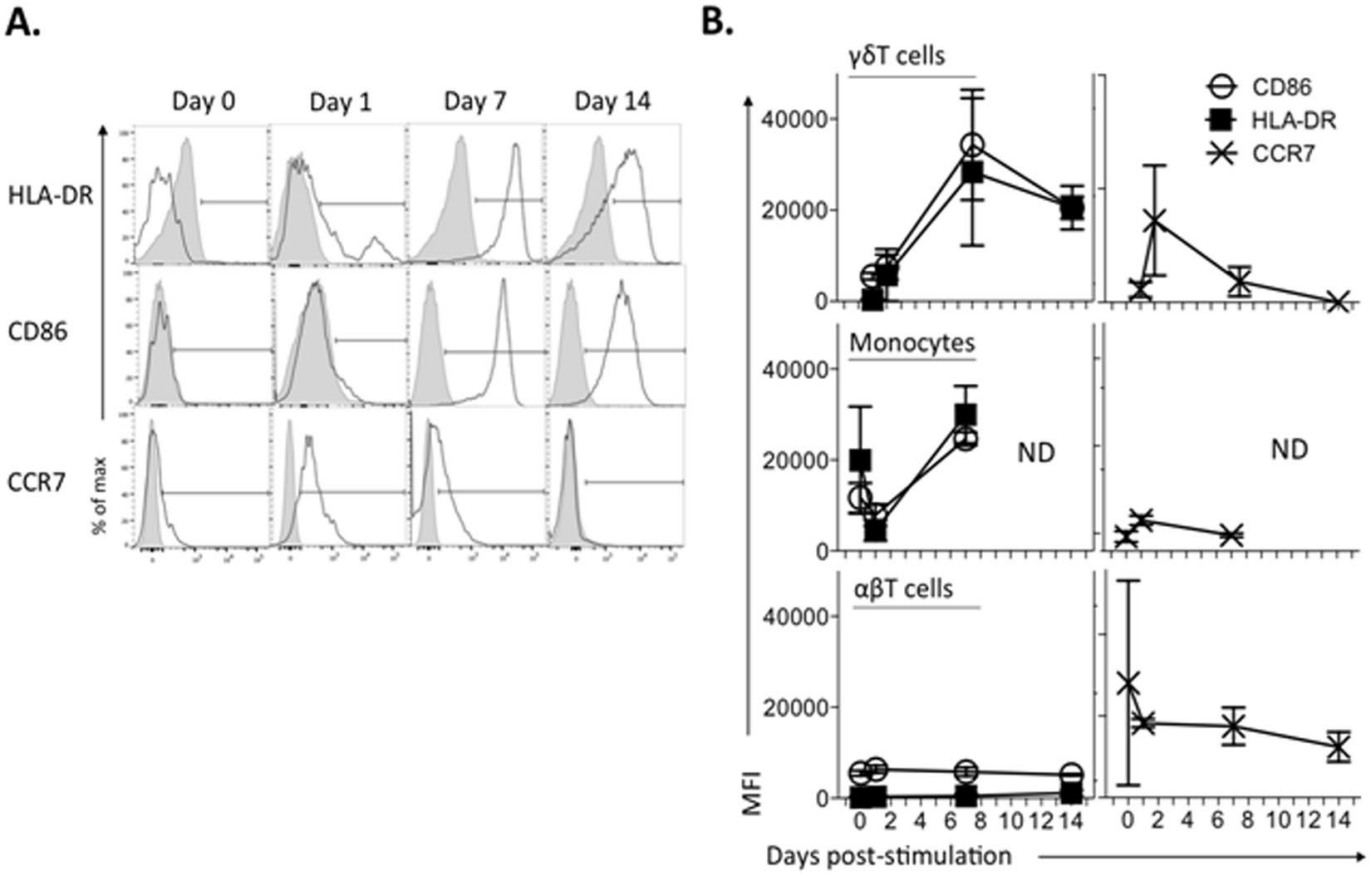
γδT cell acquisition of phagocytic capacity is concurrent with sustained upregulation of cell surface HLA-DR and CD86, but not CCR7. Freshly-isolated PBMC (n = 5) were expanded for 14 days with irradiated *E. coli*, and stained for cell surface HLA-DR, CD86 and CCR7 throughout expansion. (**A**) Representative stains of one donor γδT cells is shown, with specific antibody in black, unshaded, and isotype-matched control - in gray, shaded. (**B**) HLA-DR, CD86 (left column) and CCR7 (right column) MFI over expansion was compared between γδT, αβT cells and monocytes (CD3^neg^CD14^pos^ PBMC).

We hypothesized that opsonization-mediated phagocytosis is associated with acquisition of APC capabilities by γδT cells. To test this, expression of classic APC markers, HLA-DR and CD86, was investigated. *E. coli* mediated a steady increase in both markers, with a majority of expanding γδT cells developing an HLA-DR^pos^CD86^pos^ phenotype within 7 days of stimulation (Fig. 4A,B). HLA-DR and CD86 expression on expanded γδT cells was similar to that observed on freshly isolated monocytes (CD3^neg^CD14^pos^ PBMC). αβT cells remained negative for both markers throughout expansion (Fig. 4B). Following overnight stimulation with *E. coli*, γδT cells upregulated the lymphoid-homing chemokine receptor CCR7 which persisted up to 7 days of culture (Fig. 4A,B). When positive, CCR7 levels on γδT cells were similar to those on naïve, unstimulated αβT cells. (Fig. S4). As noted with phagocytic measurements, HLA-DR, CD86 and CCR7 expression on γδT cells were similar between *E. coli* and zoledronate-expanded cells (data not shown).

### γδT cells develop a pAPC phenotype whilst maintaining cytotoxicity and losing a TCR-dependent Th1 inflammatory phenotype

**Figure 5:**
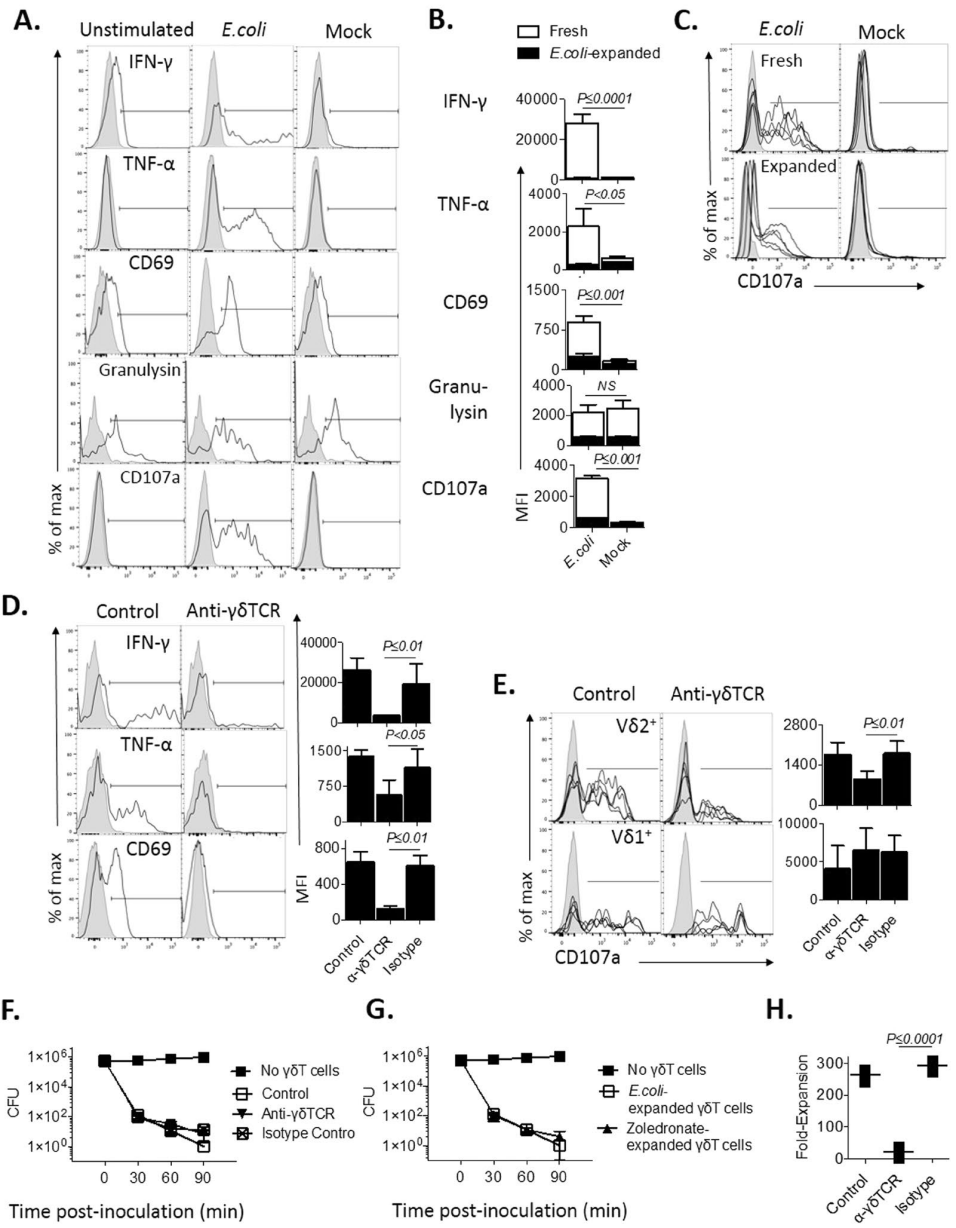
γδT cell development of a pAPC phenotype is parallel to maintenance of cytotoxicity, but leads to loss of a TCR-dependent Th1 inflammatory phenotype. Freshly-isolated PBMC (n = 5) were stimulated with *E. coli* and analyzed after overnight (16–18 h) culture or left to expand for 14 days, and re-stimulated with *E. coli* overnight on day 14. γδT cell accumulation of intracellular IFN-γ, TNF-α, granulysin, and cell surface CD69 and CD107a were assessed via FACS analysis. (**A**) Freshly-isolated PBMC were analyzed unstimulated, after overnight stimulation with *E. coli* or IL-2 alone (mock). Representative stains of one donor are shown, gated on γδT cells, with specific antibody in black, unshaded, and isotype-matched control in gray, shaded. (**B**) Parameter MFI in response to *E. coli* and mock stimulation were compared between freshly-isolated and 14 day *E. coli*-expanded γδT cells. (**C**) Stains of five representative donors are shown for cell surface CD107a in response to *E. coli* or mock stimulation, comparing fresh and *E. coli*-expanded γδT cells. (**D**) Freshly-isolated PBMC were cultured for 2 h with blocking anti-γδTCR mAb or isotype-matched control. Representative IFN-γ, TNF-α and CD69 staining of one donor, gated on γδT cells, are shown on the left. A compilation of donor parameter MFI is shown on the right. (**E**) The effect of anti-γδTCR mAb on fresh γδT cell surface CD107a is shown for five representative donors, comparing Vδ1^+^ and Vδ2^+^ cells. (**F**) *E. coli*-expanded, FACS-purified γδT cells were pre-blocked with anti-γδTCR mAb, isotype control or normal media (control) prior to co-culture with live *E. coli*. At designated time points the co-culture was lysed in H_2_O, and plated on agar for overnight growth; resulting *E. coli* CFU were counted. (**G**) *E. coli* and zoledronate-expanded, FACS-purified γδT cells were co-cultured with live *E. coli*. Remaining *E. coli* CFU were counted. (**H**) Proliferation in response to *E. coli* stimulation was observed in γδT cells pre-blocked with anti-γδTCR mAb.

To assess the γδT cell cytokine and cytotoxic profile concurrent with the acquisition of a pAPC phenotype, freshly isolated PBMC were cultured with or without UV-irradiated *E. coli* at MOI 10 and either analyzed after overnight (16–18 h) culture or left to expand in IL-2-supplemented media for 14 days, and re-stimulated with *E. coli* overnight on day 14. *E. coli* mediated potent upregulation of cell surface CD69 and CD107a, as well as marked accumulation of intracellular IFN-γ and TNF-α after overnight stimulation of γδT cells within freshly isolated PBMC, which was not seen when PBMC were mock stimulated with IL-2 media alone (representative donor data is shown in Fig. 5A). These effector responses exhibited high consistency between different donors (Fig. S5A and B). While a majority of γδT cells were IFN-γ^pos^CD107a^pos^, a significant fraction exhibited a single positive IFN-γ^pos^ or CD107a^pos^ phenotype (Fig. S5C); these were later found to be Vδ2^+^ and Vδ1^+^ cells, respectively (Fig. S5E). No IL-17 or IL-10 production could be detected by FACS or ELISA (data not shown). Approximately 65% of unstimulated γδT cells were granulysin^pos^, and this proportion did not alter significantly during mock or *E. coli* co-culture (Fig. 5A,B). αβT cells exhibited a low degree of CD107a-mediated cytotoxic degranulation in response to *E. coli* stimulation, but not upregulation of CD69, IFN-γ or TNF-α (Fig. S5D). Whilst Vδ2^+^ and Vδ1^−^Vδ2^−^ γδT cell populations were significant cytokine producers, Vδ1^+^ cells exhibited a significantly more potent cytotoxic response as measured by granulysin and CD107a expression (Fig. S5E). CD69 expression was markedly higher on Vδ2^+^ cells compared to other γδT cell subsets (Fig. S5E).

Following expansion with *E. coli*, γδT cells exhibited a significantly decreased cytokine (IFN-γ, TNF-α) response to *E. coli*, and decreased CD69 expression compared to that observed in freshly isolated γδT cells. IL-17, IL-10 or TGF-β were undetectable in *E. coli*-expanded γδT cells (data not shown). *E. coli*-expanded γδT cells did, however, continue to exhibit CD107a-mediated cytotoxic degranulation and presence of granulysin, albeit at lower levels compared to freshly isolated γδT cells (Fig. 5B). These observations may partially be attributed to the loss of the highly cytotoxic Vδ1^+^ subset after expansion (Fig. 5E). Interestingly, γδT cell expansion with zoledronate resulted in a similar loss of cytokine production capacity post-expansion, whether stimulated with *E. coli* or re-stimulated with zoledronate (data not shown). The decrease in cytokine production was not associated with a shift in memory phenotype or exhaustion marker expression (Fig. S6A), nor could it be attributed to the absence of non-T cell “helper” cells in expanded PBMC (Fig. S7). We, thus, concluded that the altered cytokine profile of expanded versus freshly isolated γδT cells represents a true phenotypic shift.

Exposure to anti-γδTCR mAb prior to co-culture of freshly-isolated PBMC with *E. coli* led to abrogation of CD69 upregulation, cytokine production (Fig. 5D) and a decrease in the double positive IFN-γ^pos^CD107a^pos^ but not in single positive CD107a^pos^ γδT cell populations (Fig. S2C). The decrease in IFN-γ^pos^CD107a^pos^ γδT cells was subset-specific, as only Vδ2^+^ but not Vδ1^+^ γδT cell cytotoxic degranulation was sensitive to a pre-blocked TCR (Fig. 5E). To evaluate direct cytotoxicity against *E. coli*, FACS-sorted *E. coli-*expanded γδT cells (predominantly with a Vδ2^+^ TCR) were pre-treated with blocking anti-γδTCR mAb and exposed to live bacteria; cytotoxicity was inferred from residual colony numbers on agar plates. Remarkably, *E. coli* colony forming unit (CFU) count declined by >90% within 30 minutes; this effect was not dependent upon γδTCR engagement (Fig. 5F). The high efficiency of bactericidal activity was observed for both *E. coli* and zoledronate-expanded γδT cells (Fig. 5G). Although γδTCR blocking showed no impact on bactericidal function, it completely blocked γδT cell proliferation in response to *E. coli* (Fig. 5H).

## Discussion

Recent studies have attested to the pAPC capacity of peripheral human γδT cells. Vγ9Vδ2T cells are able to process and present peptide antigens by MHC class II in a ‘professional’ way to naïve CD4+ T cells^22^, and to cross-present antigens on MHC class I to CD8+ T cells^31^, both reminiscent of myeloid DC. We have previously shown that, following short term activation, freshly isolated human γδT cells can phagocytose bacteria and synthetic beads, and subsequently process and present associated antigens to αβT cells^23, 24^. Further support for these observations was recently provided in a study investigating phagocytosis of *Listeria monocytogenes* by peripheral human γδT cells^16^. In parallel, numerous clinical studies indicate that human peripheral γδT cells expand significantly and transiently, and acquire the above-described pAPC features following bacterial and parasitic infections^4, 18–21, 32^. We have bridged these *in vitro* and *in vivo* clinical observations of human γδT cell functions using an *ex vivo* model system, where PBMC were cultured with *E. coli* to reflect features of *in vivo* acute bacteremia. In particular, we attempted a reconciliation of the plethora of described human γδT cell functions, ranging from IFN-γ production and cytotoxicity to phagocytosis and pAPC functions. We evaluated direct cytotoxicity, TCR dependency, and subtype specificity of these functions in order to understand whether γδT cells are able to kill directly their own microbial targets for uptake and subsequent processing, and what relationship this bears to their pAPC phenotype.

*E. coli* exposure led to potent TCR-dependent freshly-isolated γδT cell IFN-γ and TNF-α production, as well as substantial cytotoxic degranulation and bacterial killing. The ensuing, primarily Vγ9Vδ2, γδT cell proliferation/expansion was marked by concomitant upregulation of cell surface HLA-DR and CD86, and an increase in TCR-dependent bacterial phagocytic activity which was markedly enhanced by IgG opsonisation. Importantly, phagocytosis was accompanied by acidification, indicating delivery of target to the lysosomal compartment. The latter process was not seen during co-culture with αβT cells. Development of γδT cell pAPC phenotype was accompanied by a loss of cytokine production while maintaining cytotoxic degranulation and bactericidal activity. Curiously, purified expanded Vδ2^+^ cells exhibited high bactericidal activity despite decreased CD107a-mediated cytotoxic degranulation and blocking of the TCR (Fig. 5C,E and F). This may be attributable to the high efficiency of γδT cell CD107a-mediated *E. coli* killing, whereupon low level degranulation is sufficient to significantly decrease bacterial viability, or may allude instead to further CD107a-independent bactericidal mechanisms.

Overall, no significant difference in *E. coli versus* zoledronate expanded γδT cell function was observed. TCR sequencing and spectratyping revealed that TCR repertoires were strikingly similar, while differing significantly from fresh, unexpanded γδT cells, consistent with focusing of the repertoire on a common set of TCR ligands. We conclude that peripheral human Vγ9Vδ2 γδT cells transition from early TCR-dependent IFN-γ^pos^TNF-α^pos^, cytotoxic responders to TCR-dependent IFN-γ^neg^TNF-α^neg^, cytotoxic, phagocytic pAPCs. We were intrigued to discover that both uptake and acidification of opsonized *E. coli* by *E. coli*-expanded γδT cells was significantly inhibited by a γδTCR-blocking antibody (Fig. 3A); similar, although less marked, blocking was observed in zoledronate-expanded γδT cells (Fig. 3B). In addition, γδT cell uptake of material was inhibited by CyD, indicating a requirement for rearrangement of the actin cytoskeleton, as described in macrophages and DC^33–35^. A previous study demonstrated that TCR internalization by Jurkat T cells involved phagocytosis of MHC-containing membrane patches originating from an immunological synapse. In this αβT cell line the phagocytosed material was reported to be re-routed to the membrane rather than subjected to acidification and antigen processing^36^. Our observations support this published data as the subpopulation of residual αβT cells following *E. coli* stimulation acquired minor uptake but no acidification of internalized material (Fig. 2F,H). It is possible that some γδT cell phagocytosis involves a similar mechanism, recruiting phagocytising machinery to the immune synapse.

Given our data comparing the development of this phenotype in *E. coli versus* zoledronate-expanded γδT cells, we suggest that these results altogether may indicate one of the following mechanisms of TCR involvement in uptake: i) the γδTCR engages *E. coli* and beads directly, ii) *E. coli* and opsonized beads stress PBMC sufficiently to lead to the upregulation of stress markers, such as BTN3A/CD277^26^, which then provide a stimulatory signal to the γδTCR, iii) tonic TCR signaling is required for γδT cell phagocytosis, iv) interaction between TCR and FcγR, which may be disrupted by γδTCR blocking, is necessary for productive engagement of phagocytic machinery. Comprehensive further study is needed to determine the exact involvement of the TCR in γδT cell phagocytosis and further effector functions, and will benefit particularly from the examination of the role of BTN3 molecules in these processes.

It has been proposed that zoledronate activates γδT cells by causing accumulation of endogenous pyrophosphates and, consequently, conformational changes in butyrophilin molecules such as BTN3A^26^. Like zoledronate, *E. coli* too causes accumulation of endogenous pyrophosphates (IPP), which then serves to drive activation and proliferation of Vγ9Vδ2 T cells^18, 37^. *E. coli* further expresses HMBPP, a known inducer of BTN3A/CD277 conformational changes suggesting that the signal recognized by the Vγ9Vδ2 TCR is the same following IPP and HMBPP stimulation^38^. This may be a decisive factor in producing the γδT cell populations so closely related in terms of CDR3 sequences and effector function we observed following PBMC stimulation with *E. coli versus* zoledronate. Related to this is the observation that the recognition of phosphoantigen signals appears to occur primarily through germline-encoded regions of the Vγ9Vδ2 TCR, and involves all CDR loops^39^. Relative to the αβTCR, there are, moreover, relatively few germline genes available for assembly of the γδTCR^40^. It has been postulated that the expansion of the Vγ9Vδ2 subset in the periphery after birth is driven by exposure to environmental microbial ligands^41^.

TCR-engagement as a pre-requisite of phagocytosis (and other pAPC functions) suggests careful regulation of the Vγ9Vδ2 T cell compartment. Whenever an early immune response is sufficient to neutralize infection, MHC class II^pos^CD86^pos^ Vγ9Vδ2 cells may be prevented from posing an unnecessary inflammatory threat by amplifying responses further through downregulation of their early cytokine responsiveness. The requirement for opsonization of a target may be a further safety feature^23^. This could be operative at two levels: i) as herein, when opsonizing with isotype-switched target-specific IgG, ii) in a ‘naïve’ non-immune situation, where natural antibodies (NAb) of different isotype, including IgG, may be involved. We have previously demonstrated that NAb can enhance DC uptake and antigen presentation of viruses^42^. Further study of the engagement of the Vγ9Vδ2 TCR, possibly with BTN3 targets, is likely to carry significant implications for γδT cell anti-tumor immunity by supporting the notion that stress recognition, particularly in combination with Ab-opsonisation, may be sufficient not only for killing of a tumor cell but also for uptake, processing and presentation of tumor-associated antigens^23^.

γδT cell direct killing of cellular and/or microbial targets combined with inflammatory cytokine production, followed by uptake of the target into acidifying antigen processing compartments, raises a novel paradigm. It is tantalizing to hypothesize that the combination of innate-like recognition and killing followed by myeloid cell-like phagocytosis by a lymphocyte-like cell may evolutionarily have preceded the full development of T lymphocyte-mediated adaptive immunity. Interestingly, the raised hypothesis is supported by previous studies showing that γδTCR chain genes may have preceded the development of αβTCR chain genes^43^. In addition, the existence in jawless fish of three lymphocyte-like cells expressing variable lymphocyte receptors (VLR) instead of TCR or BCR chain genes also supports this contention, since they otherwise resemble αβT﻿, γδT and B cells by the expression of other orthologous genes^44^.

## Materials and Methods

### Study design

This study was designed to test the hypothesis that human γδT cells change phenotype following expansion initiated by exposure to *E. coli*. During the study we further hypothesised and tested whether blocking of the TCR on the cells would affect the phenotypic and functional changes. Sample numbers for cell expansions were 5 for most of the experiments. This number was chosen from previous and early experience in this study of known phenotypic variations between donors, in order to accurately reflect these variations. All experiments were repeated at least once, with the exception of DNA sequencing and spectratyping. However, these were done as separate experiments and the DNA sequencing of each clonotype included in the analyses were represented by multiple individual reads. All experiments using peripheral blood-derived cells were performed in accordance with relevant guidelines and regulations, and were approved by UCL Research Ethics Committee. Informed consent was obtained from all volunteer blood donors.

### Samples and cell preparation

PBMC from healthy adult donor peripheral blood were routinely extracted via Ficoll density gradient separation. Cells were cultured in supplemented RPMI 1640 media at a density of 1.5 × 10^6^ cells/mL at 37 °C and 5% CO2. Supplemented culture media contained RPMI 1640-GlutaMax (Life Technologies), 10% foetal calf serum, 1% Penicillin/Streptomycin (Life Technologies), 10 mM HEPES buffer (Life Technologies), 1 mM Sodium Pyruvate (Life Technologies) and 1x MEM non-essential amino acids (Life Technologies). All stimulation studies, unless explicitly specified, further included 100 IU/mL recombinant human IL-2 (MACS Miltenyi); media was re-adjusted every two to three days.

### Growth and preparation of *E. coli* DH5α

*E. coli* (Thermo Fisher) were grown overnight at 37 °C shaking culture in 1 mL ampicillin (Life Technologies)-supplemented LB media (Sigma) from cryogenically preserved aliquots. Once amplified, *E. coli* culture was washed thoroughly and assessed for colony-forming unit (CFU) count via duplicate measurement of suspension optical density (OD). With the exception of killing assays, all bacteria employed in co-culture experiments were irradiated in a trans-illuminator chamber (UVITEC), equipped with eight UV-C (250–280 nm) lamps.

### PBMC stimulation with UV-irradiated *E. coli*

Freshly isolated or expanded 1.5 × 10^6^ cells/mL PBMC were co-cultured with *E. coli* (MOI 10) in supplemented RPMI 1640 media, and cultured overnight (16–18 h) or left to expand for 14 days. Re-stimulation of expanded PBMC with *E. coli* was carried out by mixing 14 day *E. coli-*expanded PBMC with irradiated *E. coli* at MOI 10. Re-stimulation of expanded PBMC with *E. coli* in the presence of freshly-isolated autologous PBMC was carried out by mixing FACS-stained 14 day *E. coli-*expanded PBMC with freshly-isolated unstained autologous PBMC at a ratio of 1:10 prior to the addition of irradiated *E. coli* at MOI 10.

### PBMC stimulation with zoledronate

Freshly isolated 1.5 × 10^6^ cells/mL PBMC were cultured in 5 μM zoledronic acid monohydrate (zoledronate; Sigma-Aldrich) in supplemented RPMI 1640 media, and cultured for 14 days. Re-stimulation of expanded PBMC with *E. coli* was carried out by mixing 14 day *E. coli-*expanded PBMC with irradiated *E. coli* at MOI 10.

### PBMC surface marker expression and intracellular cytokine staining by flow cytometry

PBMC were stained for cell viability, surface markers, intracellular cytokines and cell surface CD107a throughout stimulation and expansion as indicated in supplied commercial protocols. Intracellular cytokine and CD107a staining was carried out on overnight stimulated PBMC that were cultured for a further 4 h in the presence of monensin (BioLegend). Colour compensation was carried out using OneComp eBeads (eBioscience). FACS analysis was performed on the Becton Dickinson (BD) LSR II and data processing - on FlowJo vX.07 software. The following antibody conjugates were used in PBMC staining: CD3-PE/Dazzle594 (BioLegend; clone: UCHT1), αβTCR-PE (BioLegend; clone: IP26), αβTCR-PE/Vio770 (MACS Miltenyi; clone: BW242/412), γδTCR-PE/Vio770 (MACS Miltenyi; clone: 11F2), Vδ1-FITC (Thermo Fisher; clone: TS8.2), Vδ1-APC (MACS Miltenyi; clone: REA173), Vδ2-PerCP (Biolegend; clone: B6), Vδ2-PE (Biolegend; clone: B6), IFN-γ-PE (BioLegend; clone: B27), TNF-α-APC (BioLegend; clone: MAb11), IL-17-Brilliant Violet 605 (BioLegend; clone: BL168), CD69-PerCP (BioLegend; clone: FN50), IL-10-FITC (Affymetrix eBiosciences; clone: BT-10), granulysin-PE (BioLegend; clone DH2), CD107a-FITC (BioLegend; clone: H4A3), CCR7-PE (R&D Systems; clone: 150503), CD27-APC/Vio770 (Miltenyi Biotec; clone: M-T271), CD45RA-FITC (BioLegend; clone: HI100), HLA-DR (MHC II)-APC/Cy7 (BioLegend; clone: L243), CD86-APC (MACS Miltenyi; clone: FM95). Mouse IgG1κ of known, irrelevant, non-human specificity served as isotype control (BioLegend; clone: MG1-45). All FACS data presented subsequently is on singlet, live lymphocytes. The gating strategy employed in analysis is shown in Fig. S1.

### γδT cell sorting by flow cytometry

Day 14 expanded PBMC, with a predominantly γδT cell content, were purified further using flow sorting on the LSR II to >98% purity. Prior to the sort, PBMC were stained for expression of CD3 with CD3-PE/Dazzle594 (BioLegend; clone: UCHT1) and αβTCR with αβTCR-PE/Vio770 (MACS Miltenyi; clone: BW242/412). γδT cells were sorted for as CD3^pos^αβTCR^neg^ PBMC, gated as shown in Fig. S1. Bright fluorophores were used to maximize the clear separation of expanded αβT and γδT cells. Samples of purified γδT cells were stained as described above post-sort for further γδT cell markers including Vδ2 and γδTCR to establish purity. γδT cells were sorted into 50% foetal calf serum and cultured overnight in complete RPMI 1640 prior to use in functional assays.

### *E. coli* opsonization

*E. coli* were opsonized with commercially available highly purified anti-*E. coli* rabbit serum IgG (*Escherichia coli* BioParticles Opsonizing Reagent from Thermo Fisher) according to commercial protocol.

### Confocal microscopy imaging of γδT cell uptake of *E. coli*

Imaging was performed on a Zeiss AxioObserver LSM 710 confocal microscope. FACS-purified 14 day zoledronate-expanded γδT cells were incubated with IgG-opsonized, IPTG-inducible GFP-expressing *E. coli* (Thermo Fisher) for 60 min, placed on ice and fixed. Cells were then fluorescently labeled, deposited on cleaned coverslips and mounted on glass slides using ProLong Gold antifade mountant (Thermo Fisher) and cured in the dark at room temperature for 24 h. Images of cell conjugates were acquired with a 63× Plan-Apochromat oil objective, numerical aperture 1.4. Acquisition was optimized for subsequent deconvolution with Huygens software, using appropriate voxel sizes according to the Huygens Nyquist calculator.

### Imaging flow cytometry of γδT cell uptake of polystyrene beads

Imaging was performed on an Image StreamMark II flow cytometer (Amnis). Prior to analysis, 14 day zoledronate-expanded γδT cells were incubated with protease-sensitive DQ-Green (Thermo Fisher), BSA-labelled opsonized or non-opsonized polystyrene beads 0.5 μm or 1.0 μm in size (Polysciences) for 60 min, fixed and stained for cell surface markers. The opsonin used was Rituximab, a monoclonal, chimeric human-mouse IgG (Hoffman La Roche). The mode of opsonisation was passive adsorption of antibody to the bead, according to commercial protocol as supplied by Thermo Fisher. Post-acquisition data analysis was performed using IDEAS software (Amnis). ImageStream internalisation scores (IS) were generated by IDEAS software as described in commercially supplied protocol. Briefly, IS is defined as the ratio of fluorescence intensity inside the cell to the intensity of the entire cell. The inside versus outside of the cell is judged by application of an internal mask based on the brightfield image that covers the inside of the cell, the thickness of the cell membrane in pixels and the fluorescence channel of interest, while the external region is determined by dilating the internal mask by the membrane thickness and combining this with the object mask of the channel of interest.

### *E. coli*-FITC uptake assay

A FITC-Trypan Blue quenching assay was employed to assess PBMC uptake of *E. coli*. Briefly, UV-irradiated *E. coli* DH5α were FITC labeled by a gentle shaking in a saturated FITC isomer I (Sigma)-PBS solution for 1 h at 37 ^o^C, followed by washing prior to co-culture with fresh or expanded PBMC at MOI 10. PBMC were co-cultured in triplicate for 60 min at 37 °C in a 5% CO2 incubator. Cells were then fixed in cold fixation buffer (Biolegend) before quenching with 0.4% Trypan Blue solution (Sigma-Aldrich) to remove extracellular FITC signal. After quenching, PBMC were washed three times in a large volume of PBS and analyzed using flow cytometry, as described previously by Busetto, *et al*.^45^ Each sample of quenched PBMC-*E. coli* mixture was treated in parallel to a non-quenched sample of the same origin to ensure that quenching had taken place. A quenched PBMC sample incubated with non-FITCylated *E. coli* was used as a control for background FITC fluorescence. In order to determine the involvement of actin polymerization in *E. coli* uptake, PBMC were pre-incubated in 0.2 mM CyD (Sigma), vessel control, DMSO (Sigma), or normal media.

### *E. coli*-pHrodo acidification assay

An *E. coli* assay was employed to assess PBMC acidification of internalized bacteria according to supplied commercial protocol (available from “pHrodoTM Red and Green BioParticles® Conjugates for Phagocytosis” by Thermo Fisher Scientific). Briefly, supplied PFA-fixed, pHrodo-dyed *E. coli* (strain: K-12; product code: P35366) was re-suspended in a pH neutral isotonic buffer, HBSS (Life Technologies), and co-cultured with PBMC in triplicate in a 96-well plate for 60 min at 37 °C and 5% CO2. The media used in this assay was pre-warmed HBSS, sans IL-2. After co-culture, the culture was removed into cold fixation buffer. After fixation, PBMC were washed thoroughly and FACS stained for cell surface markers. Fixed and stained PBMC were then analyzed using flow cytometry. pHrodo dyes do not fluoresce at basic or neutral pH, but fluoresce strongly in proportion to pH drop below pH of 7. *E. coli-*pHrodo alone served as control for background pHrodo fluorescence. In order to determine the involvement of actin polymerization in bacterial acidification, PBMC were pre-incubated in 0.2 mM CyD, DMSO or normal media.

### Green fluorescent bead uptake assay

A green fluorescent bead-Trypan Blue quenching assay was employed to assess PBMC uptake of opsonized beads. Briefly, streptavidinylated (SA) ‘Dragon Green’ beads (Bangs Laboratories) were incubated with anti-SA rabbit mAb (GeneScript), washed and co-cultured in triplicate with PBMC for 60 min at 37 °C in a 5% CO2 incubator. Cells were then fixed in cold fixation buffer (Biolegend) before quenching with 0.4% Trypan Blue solution (Sigma-Aldrich) to reduce extracellular Dragon Green signal. After quenching, PBMC were washed three times in a large volume of PBS and analyzed using flow cytometry. Each sample of quenched PBMC-bead mixture was treated in parallel to a non-quenched sample of the same origin to ensure that quenching had taken place.

### γδTCR blocking

PBMC were co-incubated for 2 h with 10 μg/mL LEAF-purified anti-γδTCR mouse IgG1κ mAb (Biolegend; clone: B1), the blocking properties of which have been described by Correia, *et al*.^46 ^or isotype-matched LEAF purified mouse IgG1κ mAb of known non-human specificity (BioLegend; clone: MG1-45).

### Sequencing of γδTCR CDR3

RNA was extracted from 1 × 10^6^ freshly-isolated, 14 day *E. coli-*expanded or zoledronate-expanded PBMC. cDNA synthesis and PCR amplification of the gamma and delta chain sequences was performed using a commercial kit (Irepertoire Illumina human gamma delta kit; Cat. No. HTDGI-01-P). Amplified, barcoded fragments were sequenced on an Illumina MiSEq to a depth of 250 paired ends. Analysis was performed using a commercial platform provided by Irepertoire®.

### Spectratyping of Vγ9 and Vδ2 CDR3

Spectratyping was used to determine CDR3 lengths for functional V-gamma 9 and V-delta 2 genes. RNA was extracted from 14 day *E. coli-*expanded or zoledronate-expanded PBMC. Following this, cDNA was synthesized using High Capacity RNA to cDNA (Thermo Fisher Scientific). With cDNA as the template, TCR V-delta 2 specific forward primer (TGAAAGGAGAAGCGATCGGT) or TCR V-gamma 9 specific forward primer (TGGAATGTGTGGTGTCTGGA), and a fluorescently labeled TCR C-delta (GACAAAACGGATGGTTTGG) or TCR C-gamma (GGGGAAACATCTGCATCAAG) reverse primer were used to amplify fragments of interest by PCR. Fragments were separated by size using capillary electrophoresis and analyzed by GeneMapper v3.7 software (Applied Biosystems).

### γδT cell bactericidal activity against *E. coli*

γδT cells were purified from 14 day *E. coli-*expanded PBMC using FACS sorting to >98% purity. To mitigate the stress and potential TCR internalization in response to purification, purified γδT cells were rested overnight in supplemented, antibiotic-free RPMI 1640 media. 1.5 × 10^6^ cells/mL γδT cells were then co-cultured with live, non-irradiated *E. coli* DH5α at MOI 10 for 15, 30, 60 or 90 minutes. At designated time points, PBMC-bacterial suspension was removed in duplicate into sterile-filtered, room temperature distilled H_2_O, allowed to hypotonically lyse for 10 minutes (to account for possible uptake of bacteria by γδT cells) and then serially diluted in H_2_O to 10-6 of the original concentration. The dilution series was plated in duplicate onto LB agar plates and grown overnight. The number of colony forming units (CFU) was as a measure of bactericidal activity. To determine the involvement of the γδTCR in bacterial killing, γδT cells were pre-incubated for 2 h with anti-γδTCR mAb, isotype-matched control or normal media, as described above.

### Statistical analysis

Where relevant, acquired data was evaluated statistically with paired or unpaired t tests without assumed consistent standard deviation. Statistical significance was assessed through the Holm-Sidak method of correcting for multiple comparisons. The results referred to as “significant” further in the text entail a P value of 0.05 or lower. The statistical and graphic analysis software employed was Prism 6.0.

### Data availability statement

All data generated or analysed during this study are included in this published article (and its Supplementary Information file). The new generation sequencing datasets generated during and analysed during the current study are not publicly available due to their considerable size, but are available from the corresponding author on reasonable request.

## Electronic supplementary material

Supplementary Dataset 1

## References

[1] Xiong N, R. DH (2007). Development and selection of gamma delta T cells. Immunol. Rev..

[2] Vantourout P, Hayday A (2013). Six-of-the-best: unique contributions of γδ T cells to immunology. Nat. Rev. Immunol..

[3] Thompson, K. *et al*. In *Osteoimmunology* (ed. Choi, Y.) **658**, 11–20 (Springer US, 2009).

[4] Wang L, Kamath A, Das H, Li L, Bukowski JF (2001). Antibacterial effect of human Vγ2Vδ2 T cells *in vivo*. J. Clin. Invest..

[5] Andreu-Ballester JC (2013). Association of T Cells with Disease Severity and Mortality in Septic Patients. Clin. Vaccine Immunol..

[6] Vaccaro R, Brucella A (1993). Lymphocytes bearing the gamma delta T cell receptor in acute Brucella melitensis infection. Eur. J. Immunol..

[7] Galley HF (2015). Characterisation of gamma delta (γδ) T cell populations in patients with sepsis: Gamma delta (γδ) T cells in sepsis. Cell Biol. Int..

[8] Islam D, Christensson B (2000). Disease-dependent changes in T-cell populations in patients with shigellosis. Apmis.

[9] Ho M (1994). Polyclonal expansion of peripheral gamma delta T cells in human Plasmodium falciparum malaria. Infect. Immun..

[10] Hara T (1992). Predominant activation and expansion of V gamma 9-bearing gamma delta T cells *in vivo* as well as *in vitro* in Salmonella infection. J. Clin. Invest..

[11] Arvand M, Schneider T, Jahn H-U, Hahn H (1996). Streptococcal toxic shock syndrome associated with marked γδ T cell expansion: case report. Clin. Infect. Dis..

[12] Schneider T (1997). The Number and Proportion of V#x003B4;9Vδ2 T Cells Rise Significantly in the Peripheral Blood of Patients After the Onset of Acute Coxiella burnetii Infection. Clin. Infect. Dis..

[13] Kroca M, Johansson A, Sjostedt A, Tarnvik A (2001). Vγ9Vδ2 T Cells in Human Legionellosis. Clin. Vaccine Immunol..

[14] Pinheiro MB (2012). CD4-CD8-αβ and γδ T Cells Display Inflammatory and Regulatory Potentials during Human Tuberculosis. PLoS ONE.

[15] Matsushima, A. *et al*. Early activation of γδ T lymphocytes in patients with seere systemic inflammatory response syndrome: *Shock* **22**, 11–15 (2004). 10.1097/01.shk.0000129203.84330.b3 15201695

[16] Zhu, Y. *et al*. Human γδ T cells augment antigen presentation in Listeria Monocytogenes infection. *Mol. Med* (2016). 10.2119/molmed.2015.00214 PMC5193461 27652377

[17] Venet F (2005). Both percentage of γδ T lymphocytes and CD3 expression are reduced during septic shock: *Crit*. Care Med..

[18] Kistowska M (2008). Dysregulation of the host mevalonate pathway during early bacterial infection activates human TCR γδ cells. Eur. J. Immunol..

[19] Kersten CM, McCluskey RT, Boyle LA, Kurnick JT (1996). *Escherichia coli* and Pseudomonas aeruginosa induce expansion of V delta 2 cells in adult peripheral blood, but of V delta 1 cells in cord blood. J. Immunol..

[20] Van Rhijn I (2003). Expansion of human gammadelta T cells after *in vitro* stimulation with Campylobacter jejuni. Int. Immunol..

[21] Poquet Y (1998). Expansion of Vγ9Vδ2 T Cells Is Triggered byFrancisella tularensis-Derived Phosphoantigens in Tularemia but Not after Tularemia Vaccination. Infect. Immun..

[22] Brandes M, Willimann K, Moser B (2005). Professional antigen-presentation function by human γδ T cells. Science.

[23] Himoudi N (2012). Human γδ T Lymphocytes Are Licensed for Professional Antigen Presentation by Interaction with Opsonized Target Cells. J. Immunol..

[24] Wu Y (2009). Human γδT Cells: A Lymphoid Lineage Cell Capable of Professional Phagocytosis. J. Immunol..

[25] Feurle J (2002). *Escherichia coli* Produces Phosphoantigens Activating Human γδT Cells. J. Biol. Chem..

[26] Kondo M (2011). Expansion of Human Peripheral Blood γδ T Cells using Zoledronate. J. Vis. Exp..

[27] Paul D (2013). Phagocytosis Dynamics Depends on Target Shape. Biophys. J..

[28] Russell DG, VanderVen BC, Glennie S, Mwandumba H, Heyderman RS (2009). The macrophage marches on its phagosome: dynamic assays of phagosome function. Nat. Rev. Immunol..

[29] Tollis, S., Dart, A. E., Tzircotis, G. & Endres, R. G. The zipper mechanism in phagocytosis: energetic requirements and variability in phagocytic cup shape. *BMC Syst. Biol*. **4** (2010). 10.1186/1752-0509-4-149 PMC2991294 21059234

[30] Tse SML (2003). Differential Role of Actin, Clathrin, and Dynamin in Fc Receptor-mediated Endocytosis and Phagocytosis. J. Biol. Chem..

[31] Brandes M (2009). Cross-presenting human γδ T cells induce robust CD8+ αβ T cell responses. Proc. Natl. Acad. Sci..

[32] Ness-Schwickerath KJ, Morita CT (2011). Regulation and function of IL-17A- and IL-22-producing γδ T cells. Cell. Mol. Life Sci..

[33] Cooper JA (1987). Effects of cytochalasin and phalloidin on actin. J. Cell Biol..

[34] Elliott JA, Winn WC (1986). Treatment of alveolar macrophages with cytochalasin D inhibits uptake and subsequent growth of Legionella pneumophila. Infect. Immun..

[35] Rubartelli A, Poggi A, Zocchi MR (1997). The selective engulfment of apoptotic bodies by dendritic cells is mediated by the αvβ3 integrin and requires intracellular and extracellular calcium. Eur. J. Immunol..

[36] Martínez-Martín N (2011). T cell receptor internalization from the immunological synapse is mediated by TC21 and RhoG GTPase-dependent phagocytosis. Immunity.

[37] Davey MS (2011). Human Neutrophil Clearance of Bacterial Pathogens Triggers Anti-Microbial γδ T Cell Responses in Early Infection. PLoS Pathog..

[38] Wang H (2013). Butyrophilin 3A1 Plays an Essential Role in Prenyl Pyrophosphate Stimulation of Human V 2V 2 T Cells. J. Immunol..

[39] Wang H, Fang Z, Morita CT (2010). Vγ2Vδ2 T Cell Receptor Recognition of Prenyl Pyrophosphates Is Dependent on All CDRs. J. Immunol..

[40] Porcelli S, Brenner MB, Band H (1991). Biology of the Human γδT-Cell Receptor. Immunol. Rev..

[41] Parker CM (1990). Evidence for extrathymic changes in the T cell receptor gamma/delta repertoire. J. Exp. Med..

[42] Dürrbach A, Baple E, Preece AF, Charpentier B, Gustafsson K (2007). Virus recognition by specific natural antibodies and complement results in MHC I cross-presentation. Eur. J. Immunol..

[43] Richards MH, Nelson JL (2000). The evolution of vertebrate antigen receptors: a phylogenetic approach. Mol. Biol. Evol..

[44] Hirano M (2013). Evolutionary implications of a third lymphocyte lineage in lampreys. Nature.

[45] Busetto S, Trevisan E, Patriarca P, Menegazzi R (2004). A single-step, sensitive flow cytofluorometric assay for the simultaneous assessment of membrane-bound and ingestedCandida albicans in phagocytosing neutrophils. Cytometry.

[46] Correia DV (2011). Differentiation of human peripheral blood Vδ1 + T cells expressing the natural cytotoxicity receptor NKp30 for recognition of lymphoid leukemia cells. Blood.

