# Supplementary material for: E. coli promotes human Vγ9Vδ2 T cell transition from cytokine-producing bactericidal effectors to professional phagocytic killers in a TCR-dependent manner
Source: Sci Rep. 2017 Jun 5;7:2805. doi: 10.1038/s41598-017-02886-8 (PMC5459831; doi:10.1038/s41598-017-02886-8)
Supplement: Supplementary file 1 — Supplementary Dataset 1 [file 41598_2017_2886_MOESM1_ESM.doc]

***E.* *coli* promotes transition of human Vγ9Vδ2 T cells from cytokine-producing bactericidal effectors to professional phagocytic killers in a TCR-dependent manner**

**Authors:** M. Barisa^1^, A. M. Kramer^1^, Y. Majani^2^, D. Moulding^2^, L. Saraiva^1^, M. Bajaj-Elliott^1*^, J. Anderson^2*^, K. Gustafsson^1*^

**Affiliations:**

^1^ Infection, Immunity and Inflammation Program; UCL Great Ormond Street Institute of Child Health; 30 Guilford Street, London, WC1A 1EH, United Kingdom.

^2^ Developmental Biology and Cancer Program; UCL Great Ormond Street Institute of Child Health; 30 Guilford Street, London, WC1A 1EH, United Kingdom.

*Correspondence to: E-mail: [k.gustafsson@ucl.ac.uk](mailto:k.gustafsson@ucl.ac.uk) (K.G.); [j.anderson@ucl.ac.uk](mailto:j.anderson@ucl.ac.uk) (J.A.); [m.bajaj-elliott@ucl.ac.uk](mailto:m.bajaj-elliott@ucl.ac.uk) (M.B-E).

**Supplementary Materials:**

Figs. S.1. to S.7

Fig. S.1.


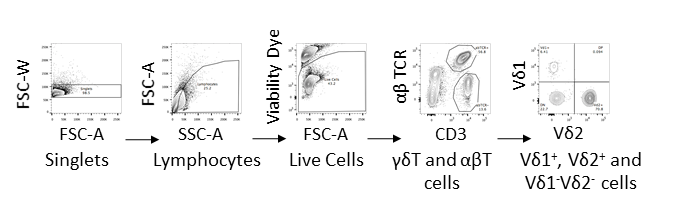


*Gating strategy.* All FACS data shown for γδT cells were gated on singlet lymphocyte live CD3^+^αβTCR^-^ and Vδ chain-determined subsets thereof. αβT cells are designated as singlet lymphocyte live CD3^+^αβTCR^+^ cells, respectively.

Fig. S.2.


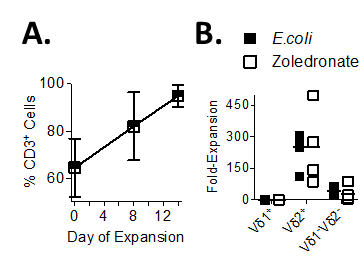


*Fresh PBMC stimulation with E.coli and zoledronate leads to preferential Vδ2^+^ γδT cell expansion****.*** Freshly-isolated PBMC (n=5) were stimulated with irradiated *E.coli* at MOI 10 and left to expand in IL-2-supplemented media for 14 days. Rates of expansion were compared between Vδ1^+^, Vδ2^+^ and Vδ1^-^Vδ2^-^ γδT cell subsets. (**A**) The proportion of CD3^+^ cells of total live PBMC was tracked over 14 days of expansion with *E.coli*. (**B**) Vδ1^+^, Vδ2^+^ and Vδ1^-^Vδ2^-^ γδT cells expansion was assessed via Trypan Blue exclusion in combination with FACS analysis.

Fig. S.3.

**
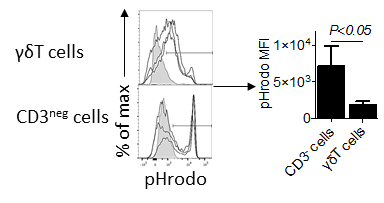
**

*Expanded γδT cells acidify bacteria with different dynamics than CD3^neg^ fresh PBMC.* Freshly-isolated or *E.coli-*expanded PBMC (n=5) were stained for cell surface markers and incubated with IgG-opsonized pHrodo-*E.coli* for 60min, and analysed via FACS. Representative stains show pHrodo in black, unshaded and a pHrodo-bacteria only control in gray, shaded. *E.coli-*pHrodo fluorescence was compared between expanded γδT cells and fresh CD3^neg^ cells. Three representative donor stains are shown on the left, and a compilation of donor MFI– on the right.

Fig. S.4.

**
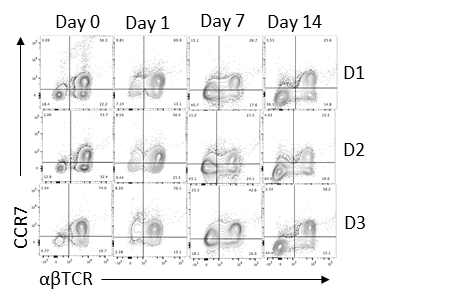
**

*αβT, but not γδT cells, sustain CCR7 expression during expansion with E.coli*. Freshly-isolated PBMC (n=5) were expanded for 14 days with irradiated *E.coli,* and stained for cell surface CCR7 throughout expansion**.** Representative 3 donors stains are shown, gated on CD3^+^ cells within expanding PBMC.

Fig. S.5.

**
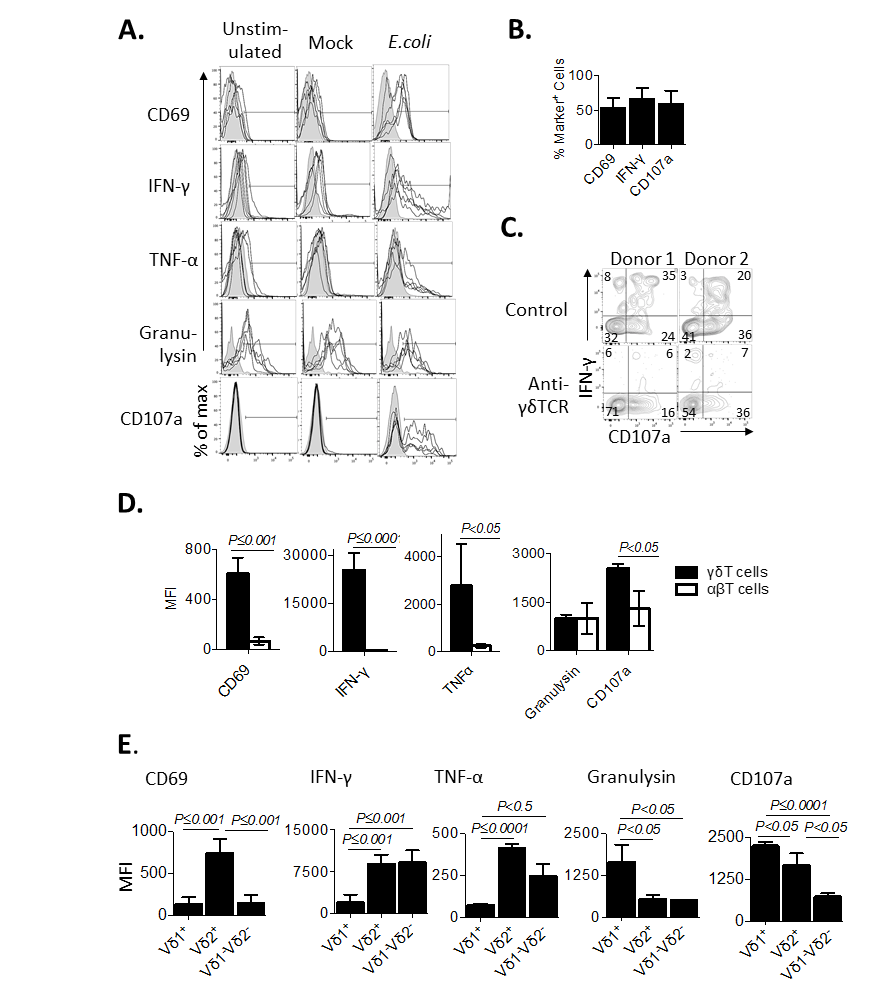
**

*Fresh PBMC stimulation with E.coli leads to preferential, subset-specific γδT cell activation and effector responses.* Freshly-isolated PBMC (n=5) were stimulated with irradiated *E.coli* at MOI 10 and either analysed after overnight (16-18h) culture or left to expand in IL-2-supplemented media for 14 days. Responses were compared between Vδ1^+^, Vδ2^+^ and Vδ1^-^Vδ2^-^ γδT cell subsets. (**A**) Shown are representative five donor sample stains of unstimulated, mock (IL-2 only) or *E.coli-*stimulated PBMC. The parameters examined are γδT cell surface CD69 and CD107a, as well as intracellular IFN-γ, TNF-α and granulysin (marker in black, unshaded overlaid with isotype control in gray, shaded). (**B**) The proportion of CD69^pos^, IFN-γ^pos^ and CD107a^pos^ cells was examined in γδT cells of overnight *E.coli-*stimulated PBMC. (**C**) Two representative donor stains are shown, indicating *E.coli-*stimulated γδT cell IFN-γ and CD107a expression with or without pre-blocking of the γδTCR with mAb. (**D**) The MFI of cell surface CD69, CD107a, as well as intracellular IFN-γ, TNF-α and granulysin were compared in γδT and αβT cells of overnight *E.coli-*stimulated PBMC. (**E**) Responses were compared in Vδ1^+^, Vδ2^+^ and Vδ1^-^Vδ2^-^ γδT cells of overnight *E.coli-*stimulated PBMC.

Fig. S.6.


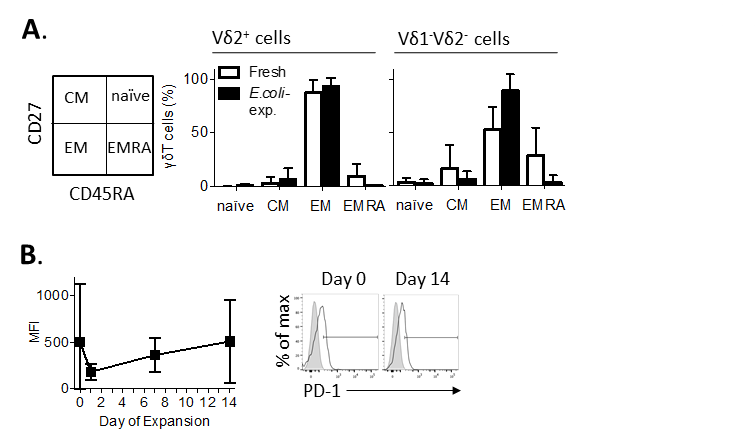


*Expansion in response to PBMC stimulation with E.coli does not lead to a significant change in γδT cell memory phenotype or PD-1 expression.* (**A**) γδT cell memory phenotype, as determined by cell surface expression of CD27 and CD45RA, was compared between fresh and *E.coli-*expanded Vδ2^+^ and Vδ1^-^Vδ2^-^ γδT cells (n=7). (**B**) Cell surface PD-1 MFI on γδT cell surface was tracked throughout PBMC expansion with *E.coli.* Representative donor stains are shown comparing PD-1 expression on fresh (D0) and expanded (D14) γδT cells with PD-1 in black, unshaded, overlaid with isotype control in gray, shaded.

Fig. S.7.

**
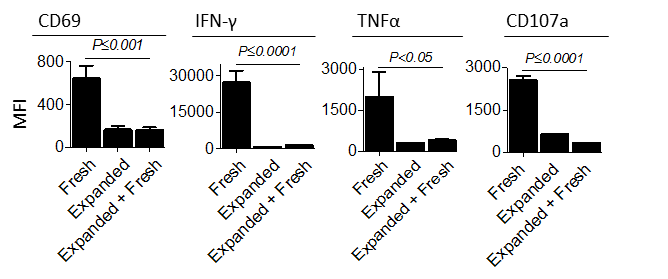
**

*γδT cell primary response effector phenotype is not rescued by the addition of fresh PBMC to expanded γδT cell culture.* 14 day *E.coli-*expanded γδT cell culture (n=5) was stained for cell surface markers and supplemented with freshly-isolated, autologous PBMC at a ratio of 1:10 prior to overnight co-culture with *E.coli* at MOI 10. γδT cell responses were compared in terms of cell surface CD69, CD107a, as well as intracellular IFN-γ and TNF-α between freshly-isolated PBMC, 14 day expanded PBMC and and 14 day expanded PBMC supplemented with freshly-isolated PBMC.
